# Conduit stability effects on intensity and steadiness of explosive eruptions

**DOI:** 10.1038/s41598-018-22539-8

**Published:** 2018-03-07

**Authors:** Álvaro Aravena, Raffaello Cioni, Mattia de’Michieli Vitturi, Augusto Neri

**Affiliations:** 1 Dipartimento di Scienze della Terra, Università di Firenze, Firenze, Italy; 2 Istituto Nazionale di Geofisica e Vulcanologia, Sezione di Pisa, Pisa, Italy

## Abstract

Conduit geometry affects magma ascent dynamics and, consequently, the style and evolution of volcanic eruptions. However, despite geological evidences support the occurrence of conduit widening during most volcanic eruptions, the factors controlling conduit enlargement are still unclear, and the effects of syn-eruptive variations of conduit geometry have not been investigated in depth yet. Based on numerical modeling and the application of appropriate stability criteria, we found out a strong relationship between magma rheology and conduit stability, with significant effects on eruptive dynamics. Indeed, in order to be stable, conduits feeding dacitic/rhyolitic eruptions need larger diameters respect to their phonolitic/trachytic counterparts, resulting in the higher eruption rates commonly observed in dacitic/rhyolitic explosive events. Thus, in addition to magma source conditions and viscosity-dependent efficiency for outgassing, we suggest that typical eruption rates for different magma types are also controlled by conduit stability. Results are consistent with a compilation of volcanological data and selected case studies. As stability conditions are not uniform along the conduit, widening is expected to vary in depth, and three axisymmetric geometries with depth-dependent radii were investigated. They are able to produce major modifications in eruptive parameters, suggesting that eruptive dynamics is influenced by syn-eruptive changes in conduit geometry.

## Introduction

**Figure 1:**
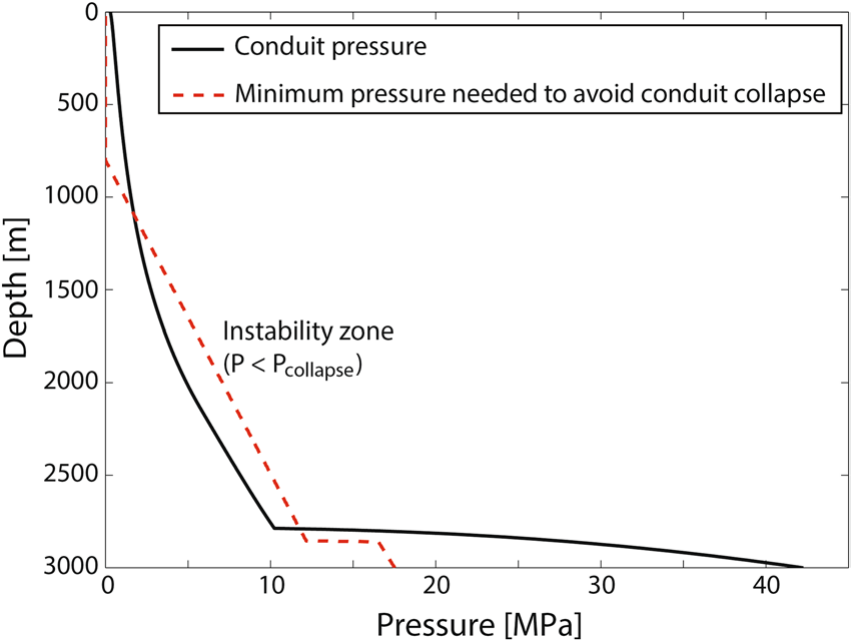
Depth versus pressure in the conduit (P(z)) and minimum pressure needed to avoid conduit collapse (P_collapse_(z)), estimated using the Mogi-Coulomb collapse criterion. The difference between both curves was employed for calculating the ‘instability index’ (max(P_collapse_(z) − P(z))), which exhibits positive values for unstable conduits and negative values for stable conduits. These results are related with a specific simulation, and are representative of mechanically unstable conduits (dacitic magma, radius of 20 m, water content of 5.5 wt.% and inlet pressure of 115 MPa).

Magma ascent dynamics during explosive eruptions is mainly controlled by the relation between magma supply and discharge rate^1,2^, which are strongly related to magma chamber conditions and volcanic conduit geometry^3^. Despite several numerical models have been employed for studying the physical conditions of ascending magmas^4–6^, they commonly assume mechanically stable geometries with constant radius in depth. Macedonio *et al*.^7^ identified four erosive mechanisms able to produce syn-eruptive changes in conduit geometry, indicating the zones where each one is probably dominant. Nonetheless, conditions for syn-eruptive mechanical stability of volcanic conduits have never been addressed in detail, nor has been studied the effect of magma rheology on them. Aravena *et al*.^8^ proposed that conduit dimensions control its mechanical stability, and a minimum radius (and hence a minimum mass flow rate) for reaching stable conditions can be computed. Under these assumptions, axisymmetric conduits with fixed radius represent a mechanically stable configuration only for a narrow range of conditions, and widening processes probably lead to geometries characterized by depth-dependent dimensions. Thus, since magma rheology controls the pressure profile along the conduit, which in turn controls the mechanical stability, important hints onto the relationships between magma composition, conduit stability and eruptive style are here derived by extending this analysis to different magma types commonly involved in large explosive events. In order to investigate this, we compare stability conditions for typical phonolitic, trachytic, dacitic and rhyolitic explosive eruptions. The most important eruptive variables and the pressure profile along the conduit (P(z)) were derived by using a 1D steady-state model^8,9^ (http://demichie.github.io/MAMMA) and, with the objective of determining the minimum pressure needed to avoid conduit collapse (P_collapse_(z)), we applied the Mogi – Coulomb collapse criterion^10^, as illustrated in Fig. 1. Additionally, we studied the expected temporal evolution of eruptions under the effects of conduit enlargement, considering trachytic and rhyolitic magmas. We also tested the consequences on the eruptive dynamics of three axisymmetric geometries with depth-dependent radii, using four different magma compositions. Finally, data from selected case studies were analyzed, for a semi-quantitative validation of modeling results.

## Results and Discussion

### Mechanical stability of volcanic conduits

**Figure 2:**
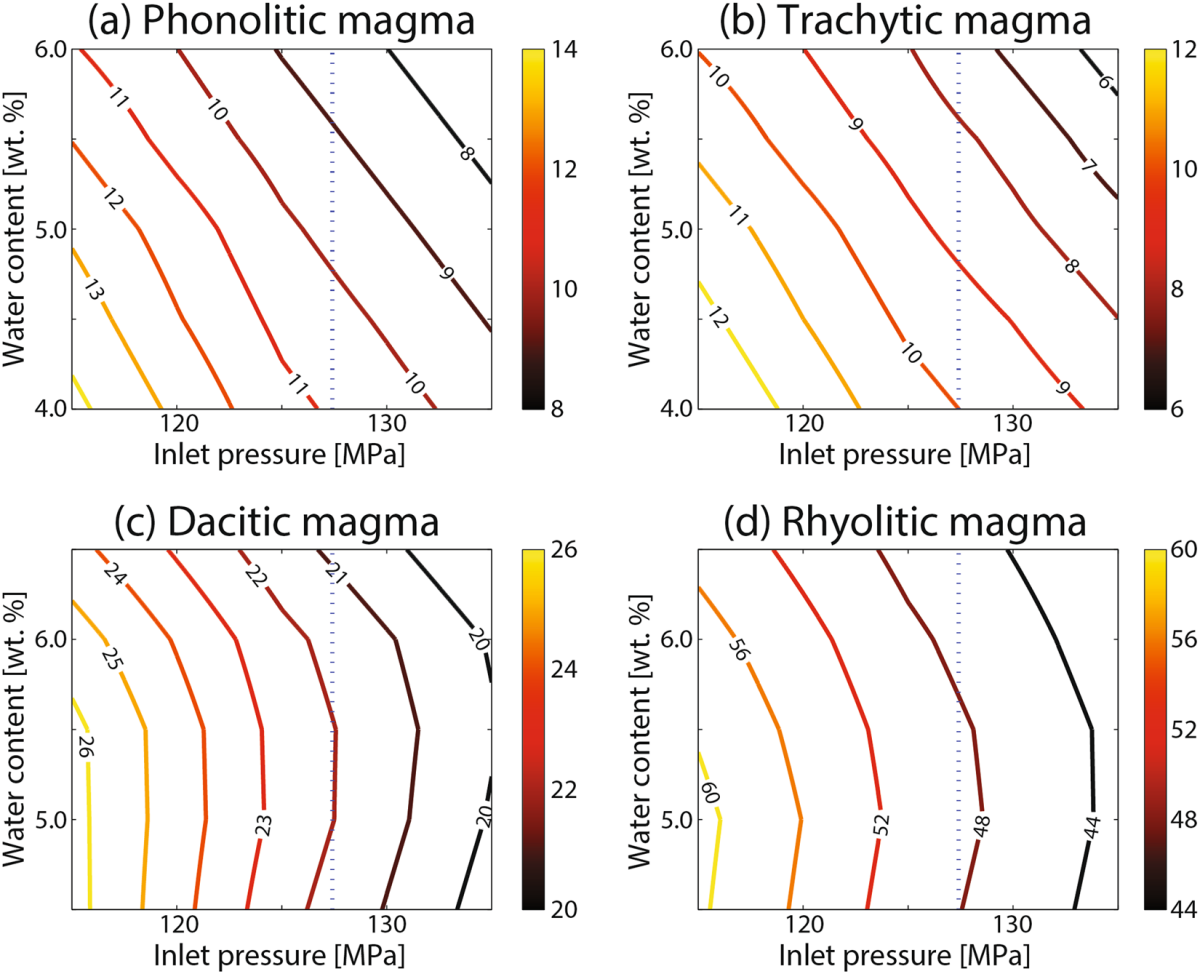
Critical radius isolines (i.e. minimum radius for a stable conduit) for different values of inlet pressure and water content. Magma compositions are indicated in titles. The differences with some results presented in Aravena *et al*.^8^ (exclusively for rhyolitic magmas) are derived from the inclusion of a more appropriate model for describing the effect of bubbles on viscosity.

In explosive eruptions, conduit collapse conditions, quantified using an ‘instability index’ defined as max(P_collapse_(z) − P(z)), are preferably reached near and above the fragmentation level, a zone characterized by an abrupt pressure drop in the ascending magma (Fig. 1)^7,8^. Consequently, instability conditions are favored by deep magma fragmentation, and a monotonic tendency is observed between instability index and conduit radius^8^. Figure 2a–d shows the isolines of the minimum radius needed for developing stable conduits (hereafter, critical radius) as a function of water content and inlet pressure, for different magma compositions.

Results indicate that magma rheology is a major controlling factor of the instability character of volcanic conduits. Indeed, in order to achieve stability conditions, dacitic/rhyolitic magmas need conduits several times wider than their phonolitic/trachytic counterparts; and significant differences are also observed between dacitic and rhyolitic melts. It is also interesting to note that, for dacitic and rhyolitic magmas, the stable conduits span over about just one order of magnitude, while they vary over about two orders of magnitude for phonolitic and trachytic magmas. In particular, for the range of input parameters here adopted and considering a 5000-m long conduit, critical radii range between 7 and 14 m for phonolitic magmas, whereas predicted critical radii for trachytic magmas vary between 6 and 13 m. Conversely, critical radii larger than 20 m and 50 m were estimated for dacitic and rhyolitic magmas, respectively. Hence, if we assume that volcanic conduits are continuously widened up to reach stability conditions, numerical simulations suggest that the ascending magma rheology controls the expected conduit dimensions and thus the resulting eruption rate. These results are consistent with the high mass discharge rates commonly observed in sustained dacitic/rhyolitic eruptions, whereas phonolitic/trachytic magmas are capable of producing sustained eruptions with narrower conduits and lower mass fluxes, as confirmed by a compilation of global data and the associated variance analysis (Supplementary Fig. S1 and Supplementary Tables S1 and S2). Indeed, statistically significant differences have been identified between the average values of eruptive column height of phonolitic/trachytic and dacitic/rhyolitic explosive events (23.6 and 30.1 km, respectively), which would be related with important differences in the corresponding mass discharge rates (~1.5·10^8^ and ~4.7·10^8^ kg/s for the average eruptive column height of phonolitic/trachytic and dacitic/rhyolitic magmas, respectively)^11^. Hence, in addition to magma source conditions (e.g. water content, inlet pressure) and the viscosity-dependent efficiency of outgassing mechanisms (i.e. permeable degassing and bubbly flow), we suggest that typical eruption rates for different magma compositions are also controlled by conduit stability and its interplay with the changing properties of ascending magmas.

**Figure 3:**
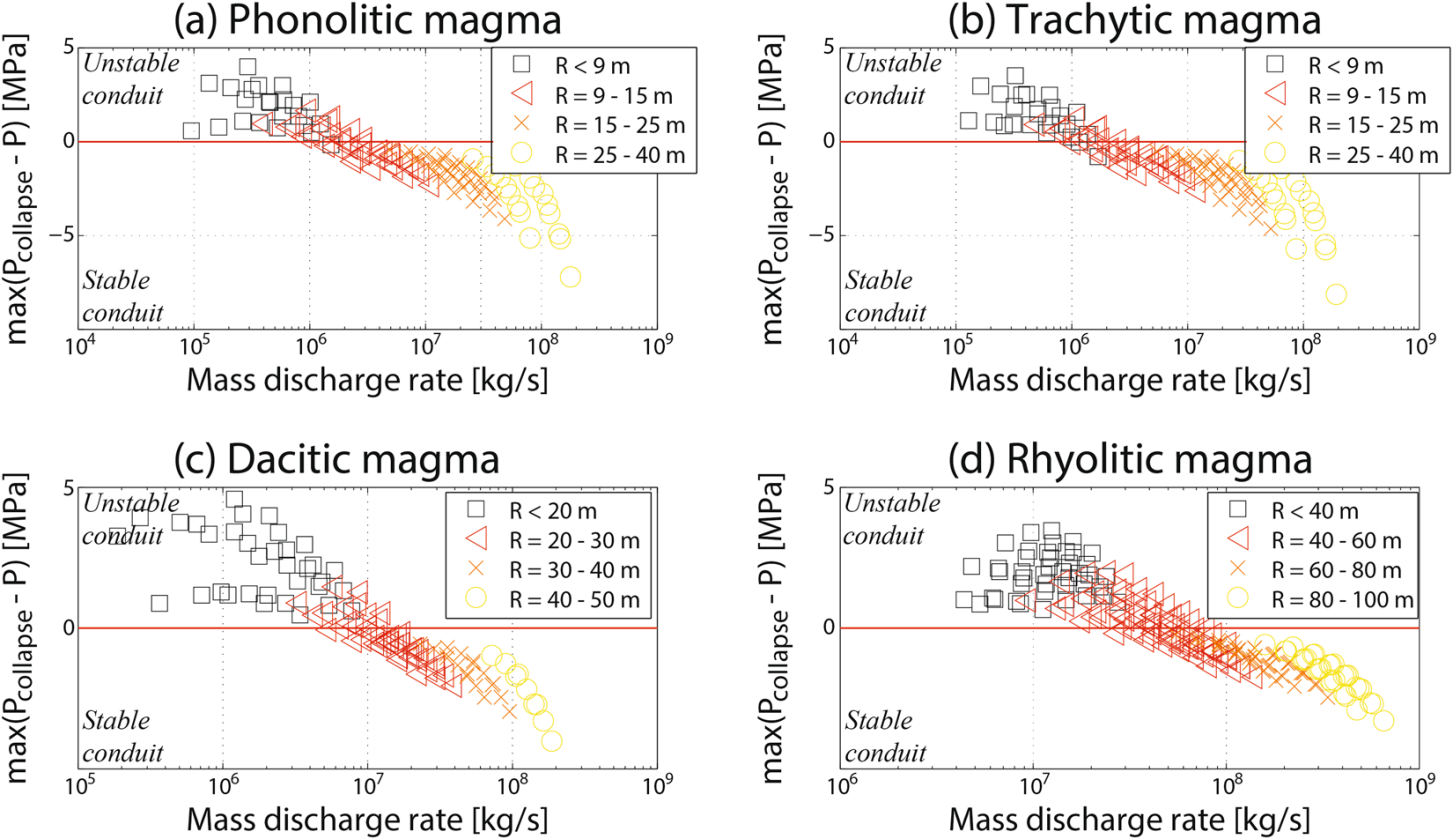
Instability index versus mass discharge rate, for simulations performed with phonolitic, trachytic, dacitic and rhyolitic magmas (in titles). We present here results related to a set of simulations with variable values of inlet pressure (115–135 MPa) and water content (4.0–6.0 wt.% for phonolitic and trachytic magmas; and 4.5–6.5 wt.% for dacitic and rhyolitic magmas).

Moreover, in order to produce a stable conduit, a minimum mass discharge rate can be derived for all the studied compositions (1·10^6^–3·10^6^ kg/s for phonolitic and trachytic magmas, 6·10^6^–2·10^7^ kg/s for dacitic magmas and 3·10^7^–8·10^7^ kg/s for rhyolitic magmas, Fig. 3). Although these results are sensitive to mechanical properties of the country rocks^12^, they suggest that the unsteady character commonly observed in low-mass flux eruptions could be produced by an unstable, narrow conduit characterized by episodic collapse events. Additionally, because differences of 1–2 orders of magnitude are observed between the estimates for the different studied compositions (from 1·10^6^ to 8·10^7^ kg/s), results suggest that the characteristic magma discharge rates needed for developing stationary explosive eruptions are highly controlled by magma rheology. Indeed, while quasi-steady, Plinian to sub-Plinian eruptions of phonolitic/trachytic magmas are often associated to intermediate values of mass discharge rate, steady dacitic/rhyolitic eruptions are generally characterized by very high magma discharge rates^13,14^, possibly stabilized by the development of wide conduits. Moreover, if collapsing conduits inhibit magma ascent, a minimum mass discharge rate for developing explosive eruptions is expected, significantly higher for dacitic/rhyolitic magmas than for their phonolitic/trachytic counterparts.

**Figure 4:**
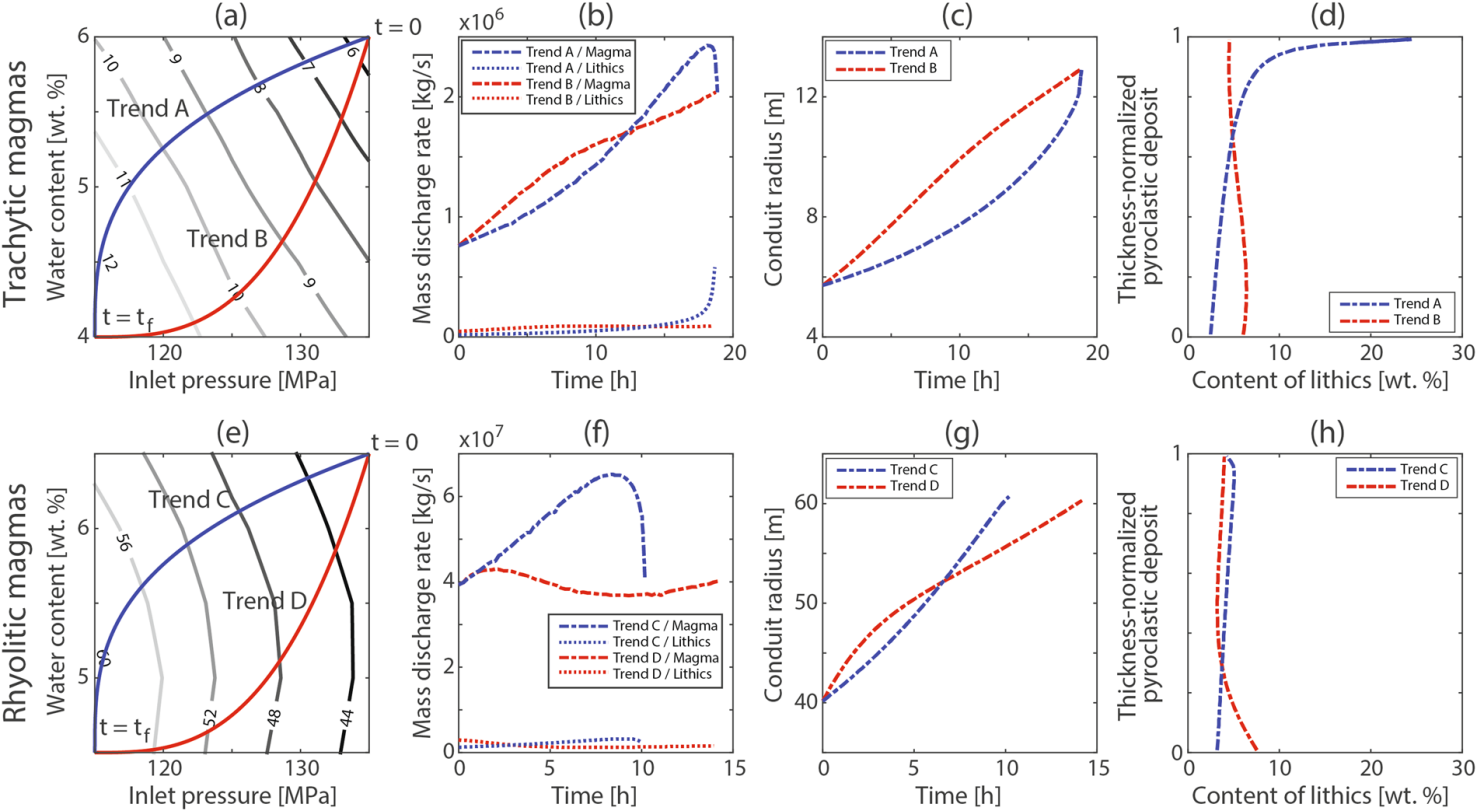
Numerical results associated to the temporal evolution of explosive events characterized by cylindrical conduits with dimensions defined by the critical radius, which varies as a function of inlet overpressure and water content. Here we consider trachytic (panels (a–d)) and rhyolitic (panels (e–h)) magmas. Each one includes two variation trends of water content as a function of inlet pressure (trends A and B for trachytic magmas, panel (a); trends C and D for rhyolitic magmas, panel (e)). Results include the temporal evolution of mass flux of magma and lithic fragments (panels (b) and (f)), temporal evolution of critical radius (panels (c) and (g)) and content of lithic fragments through the resulting pyroclastic deposit (panels (d) and (h)). For panels (d) and (h), the percentages of lithic fragments are sensitive to the total mass of eruption, but their temporal modification trends are not. These simulations assume a constant temperature of 900 °C (trends A and B) or 850 °C (trends C and D) and 5000-m long conduits. It is worth to note that the coincidence between total eruption time of simulations A and B is only a casual result derived from the nature of critical radius curves, and the end of eruptions is assumed to occur when a specific inlet overpressure is reached (in this case, −10 MPa). Please note that since relative modifications of conduit dimensions are higher for trachytic magmas in comparison to rhyolitic melts, eruption rates of trachytic magmas seem to be more sensitive to the effect of conduit stability, while rhyolitic magmas present a stronger dependence on magma source properties.

Syn-eruptive modifications of conduit geometry represent a crucial challenge in the analysis of volcanic eruptions, and the relative importance between the different erosion mechanisms raises additional questions for numerical modelling^7^. By assuming that conduit re-equilibrium to the changing magma properties may preserve nearly-axisymmetric geometries with fixed radius in depth, we can assume that volcanic conduits adopt a set of geometries whose dimensions are defined by the critical radius, which varies as a function of inlet overpressure and water content (Fig. 2). This condition is reasonable when fluid shear stress and overpressure-dominated fracture processes are enough efficient below magma fragmentation and the characteristic time for conduit collapse is sensibly lower than the timespan required for the occurrence of significant modifications in the ascending magma properties. Results indicate that temporal evolution of eruption rate is highly controlled by the balance between modifications in stability conditions of volcanic conduits (and hence conduit dimensions) and variations of source properties (e.g. water content, inlet pressure) (Fig. 4). In this sense, since relative modifications of conduit dimensions are higher for trachytic magmas in comparison to rhyolitic melts (differences of ~120% and ~50% between maximum and minimum critical radii, for the ranges considered for trachytic and rhyolitic magmas, respectively), eruption rates of trachytic magmas seem to be more sensitive to the effect of conduit stability, while rhyolitic magmas present a stronger dependence on magma source properties and their effects on the eruptive dynamics (Fig. 4b and f). Different variation trends of water content as a function of inlet pressure are also manifested in the lithic content through pyroclastic deposits, giving rise to diverse variation tendencies of the percentage of lithic fragments in the resulting pyroclastic deposits (Fig. 4c and g).

### Axisymmetric geometries with depth-dependent radius

**Figure 5:**
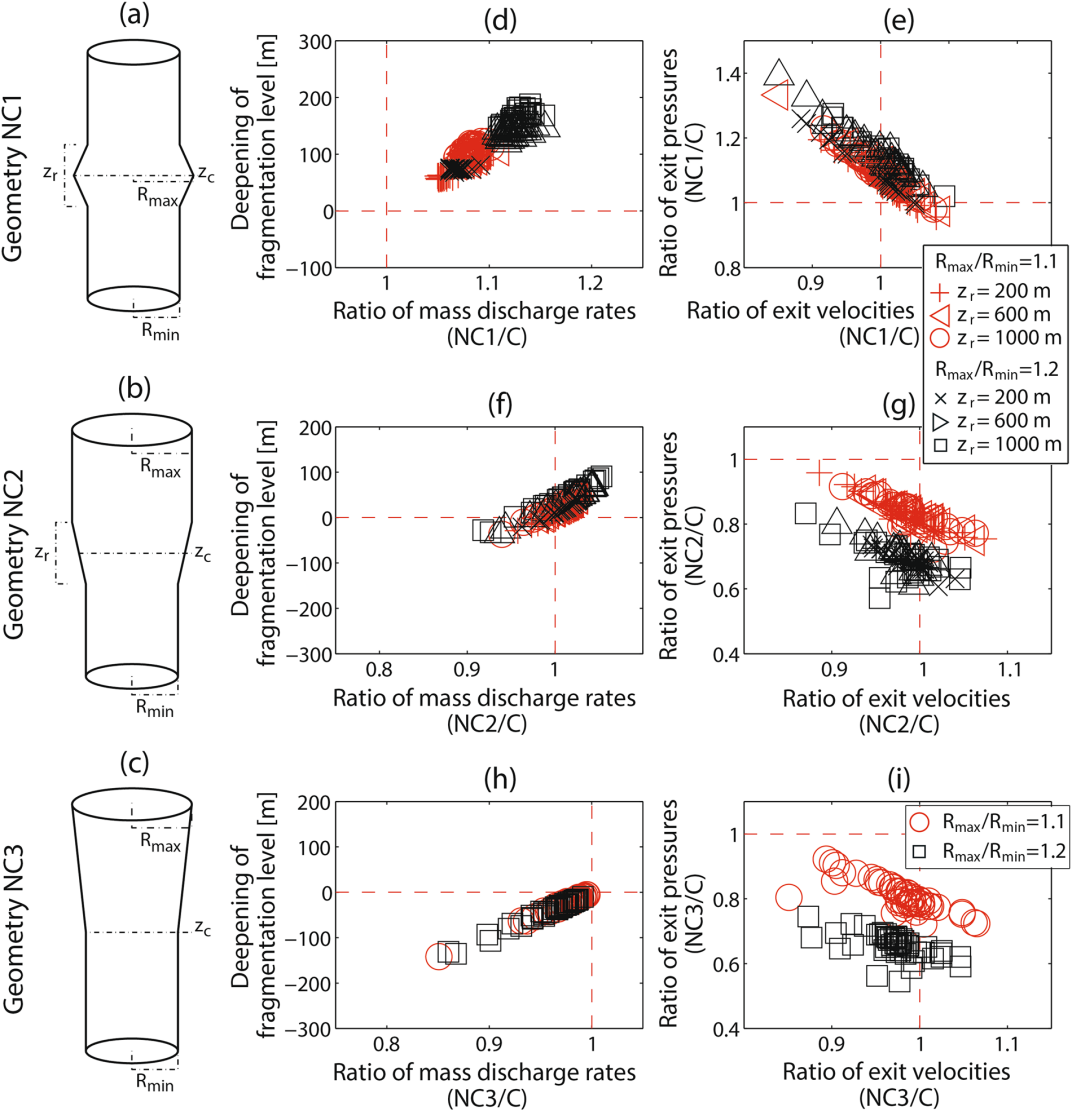
(**a**) Axisymmetric conduit with fixed radius in depth and a local enlargement along a vertical distance z_r_ (geometry NC1). (**b**) Conduit with two coaxial cylindrical portions connected by a linearly variable transitional zone of length z_r_ (geometry NC2). (**c**) Conduit with a lower axisymmetric portion with fixed radius in depth connected with the surface by a linearly enlarging zone (geometry NC3). (**d**) Ratio of mass discharge rates versus deepening of fragmentation level (NC1 versus fixed-radius equivalent case). (**e**) Ratio of exit velocities versus ratio of exit pressures (NC1 versus fixed-radius equivalent case). (**f**) Ratio of mass discharge rates versus deepening of fragmentation level (NC2 versus fixed-radius equivalent case). (**g**) Ratio of exit velocities versus ratio of exit pressures (NC2 versus fixed-radius equivalent case). (**h**) Ratio of mass discharge rates versus deepening of fragmentation level (NC3 versus fixed-radius equivalent case). (**i**) Ratio of exit velocities versus ratio of exit pressures (NC3 versus fixed-radius equivalent case). Here we present results related to rhyolitic magmas, whereas results related to dacitic, trachytic and phonolites magmas are exhibited in Supplementary Figs S2, S3 and S4, respectively.

Since stability conditions are not uniform along the conduit and the other erosive mechanisms act preferentially around and above magma fragmentation level^7,8^, preservation of cylindrical geometries with fixed radius along the conduit seems to be unlikely in natural conditions. For this reason, we tested three plausible axisymmetric conduit geometries with depth-dependent radii (Fig. 5a–c), in which the onset of conduit radius variations is concentrated around the fragmentation level of the fixed-radius equivalent case, as suggested by conduit mechanical stability.

In relative terms, variations in eruptive dynamics related to these conduit geometries exhibit similar trends for all the studied magma compositions (Fig. 5d–i and Supplementary Figs S2, S3 and S4 for comparisons). Results suggest that the evolution of the most important eruptive parameters is conditioned by the modality of conduit erosive processes. If the conduit is capable of inhibiting the collapse above the fragmentation level (i.e. producing NC1-like geometries, Fig. 5d and e), a significant increase in the mass discharge rate is expected (up to ~15%), which could be amplified by a downward migration of the fragmentation level (up to ~200 m) and, consequently, by the occurrence of instability conditions and widening processes in deeper zones of the conduit. Additionally, exit pressure tends to be higher than that observed in the fixed-radius equivalent case (in general, not higher than 30%), whereas the exit velocity changes, controlled by choked conditions at the vent, are more limited (in general, ±10%). On the contrary, if significant conduit enlargement occurs above the fragmentation level (i.e. producing NC2-like or NC3-like geometries, Fig. 5f–i), smaller modifications in magma discharge rate (in general, ±10%), fragmentation level (in general, ±100 m) and exit velocity (±10%) will occur, while a significant decrease in the exit pressure is expected (60–80% of the fixed-radius equivalent case or so).

Because of the strong contrast between the wall-rock volumes to erode for producing the different axisymmetric geometries with depth-dependent radii, important differences are expected on the composition and relative amount of lithic fragments in pyroclastic deposits. Indeed, the ratio between the mass of eroded lithic fragments and the eruption rate reveals that the expected lithic fraction with geometries NC2 and NC3 is one order of magnitude higher than that expected with geometry NC1, even though geometry NC1 produces more important effects on the eruptive dynamics. Thus, no direct relations between the observed fraction of lithic fragments in pyroclastic deposits and the magnitude of the eruptive dynamics variations are expected; and the effectiveness of wall rocks erosion processes in controlling the eruptive behavior is much higher for NC1-like geometries. Since axisymmetric geometries with depth-dependent radii do not necessarily tend to stability conditions^8^, it is not possible to suggest a continuous accommodation to stable geometric configurations. Instead, volcanic conduits would be characterized by permanent instability conditions and conduit geometry would be controlled by the characteristic times for changes in magma source properties (e.g. water content, inlet pressure) and gravitational collapse (eventually, as a function of instability degree), in addition to the effect of other erosion mechanisms. This is indirectly confirmed by the ubiquitous presence of lithic fragments throughout all the deposits of large pyroclastic eruptions.

### Analysis of selected case studies

Data from selected eruptions can be used for a semi-quantitative validation of the modeling results. The trachytic 1630 eruption of Furnas volcano (Sao Miguel, Azores) represents an appropriate case study for illustrating the relation between mass discharge rate and the unsteady character of volcanic eruptions. It was a mainly pulsating event characterized by relatively low mass discharge rates (0.2·10^6^–6·10^6^ kg/s)^15^ and fluctuations between magmatic and phreatomagmatic phases. Our results suggest that the low mass discharge rates produced unstable conditions in the conduit, generating episodic collapse events manifested in the pulsating nature of the eruption, possibly favoring the access of external water into the conduit. Indeed, since pore pressure favors conduit instabilities, collapse conditions were possibly concentrated around aquifer rocks^12^, producing magma-water interaction and the observed phreatomagmatic behavior during these pulsations. The range of mass discharge rate for this eruption is similar to that estimated for other sub-Plinian eruptions with magmas of similar rheology and characterized by an important unsteadiness^16,17^, suggesting a possible cause-effect relation with conduit stability conditions. In particular, the main part of the pyroclastic deposits related to the 512 AD phonolitic to tephri-phonolitic eruption of Vesuvius is characterized by a striking grain size alternation of fine to coarse lapilli, interpreted as the product of a highly oscillatory sub-Plinian phase^16^, followed by a pulsating phreatomagmatic phase. A similar unsteady behavior was interpreted for the initial sub-Plinian phase of the 472 AD eruption of Somma-Vesuvius (phonolitic to tephri-phonolitic magmas)^17^, with the alternating deposition of pyroclastic fall beds and pyroclastic density currents, also in this case followed by phases characterized by phreatomagmatic fragmentation.

The detailed, systematic study of the rhyolitic eruptions of the Mangaone subgroup (New Zealand)^13,14^ provides a sort of homogeneous database highly suitable for testing our results. In general, much higher eruption rates, respect to those discussed for the phonolitic/trachytic case, characterize this set of quasi-sustained Plinian eruptions, with peak mass discharge rates between 6.2·10^7^ kg/s and 3.9·10^8^ kg/s (Supplementary Table S3 and Supplementary Fig. S5) and the ubiquitous presence of lithic fragments and lithic-rich horizons in most of the studied units. Authors distinguished two situations^13,14^: (1) lithic-rich layers confined to pumice beds characterized by changes in the pumice size and/or evidences of interaction with external water, and interpreted as partial blockages of the conduit; and (2) lithic-rich layers in beds with uniform pumice size, indicating limited wall collapses, insufficient for interfering with the quasi-steady character of the Plinian eruption. The extent of conduit blockage related to wall collapse events is controlled by the collapsing volume and conduit dimensions. We suggest that, since narrow conduits produce favorable conditions to wall collapse (and consequent conduit blockage), the initial erosion rate (i.e. during inception and waxing phases) plays a crucial role in the viability of sustained Plinian eruptions. Indeed, seven units (B, C, D, E, F, G and K) out of twelve of Mangaone Subgroup are characterized by basal, lithic-rich deposits and peak mass discharge rates variable from 8.6·10^7^ up to 3.9·10^8^ kg/s. This clearly suggests that, in order to maintain a sustained flux of rhyolitic magma, an early conduit enlargement process is needed, and a consequence of this enlargement is the establishment of the high magma discharge rates typical of sustained Plinian eruptions^18^. On the other hand, the only two eruptions characterized by clear phases of collapsing column and ignimbrite deposition (units I and L), present a generally high lithic content throughout the entire sequence, suggesting that the continuous increase in conduit diameter was possibly one of the reasons for determining collapse conditions of the eruptive column. In addition, most of the units present lithic-rich beds during the waning phases and occasionally exhibit evidences of magma-water interaction^13,18^, interpreted as the result of incipient wall collapses produced by inlet pressure drops, before the total blockage of the conduit (i.e. shutdown of the eruption). Taupo pumice represents an additional case study for addressing sustained silicic eruptions^19^. In this case, the reported eruption rate is higher than 10^9^ kg/s and, in agreement with our results, it produced a sustained explosive event. Well-constrained values of total lithic content (1.8·10^12^ kg)^19^ provide some lights for estimating feeding conduit dimensions. Considering an 8000-m long axisymmetric conduit with fixed dimensions in depth, radii larger than 170 m are expected, producing a highly stable conduit according to our results. Numerical simulations suggest that such particularly large conduit diameter could be related to an efficient conduit-opening process, probably favored by magma-water interaction^12^, with significant consequences on the resulting mass discharge rate. Additional suitable information is provided by the dacitic-rhyodacitic 1932 Quizapu eruption (Chilean Andes), which produced an “exceptionally uniform fall deposit”^20^. Still, as observed in most of the rhyolitic Mangaone eruptions, also in this case the lower part of the deposit is richer in lithic fragments than the upper part, and a general (although weak) upward coarsening of the fall deposit is described. Despite the percentage of lithic fragments is low, because of the high erupted volume, it resulted in a large volume of lithic fragments^20^, implying conduit radii larger than 100 m. These conduit dimensions and the reported mass discharge rate (9·10^7^–2.5·10^8^ kg/s)^20^ are in agreement with the development of a very stable feeding conduit and a highly steady explosive eruption, manifested in the described uniformity of pyroclastic deposits^20^.

On the other hand, Inyo Craters and Mono Craters (eastern central California) provide a subset of silicic explosive eruptions characterized by relatively low eruption rates^21–23^. During the latest explosive event in the Inyo volcanic chain, ~0.13 km^3^ of rhyolitic tephra was erupted in four successive eruptions. The first two eruptions produced pulsating, sub-Plinian columns separated by a period of column collapse^22^, exhibiting mass discharge rates lower than 10^7^ kg/s, which is in agreement with the highly unstable character expected for these MDRs in rhyolitic/rhyodacitic feeding conduits. The latter two eruptions were characterized by higher MDRs (up to ~4·10^7^ and ~3·10^7^ kg/s, respectively), and the development of more sustained weak plumes, still with punctual periods of phreatomagmatic activity^22^. The transition between the second (South Deadman 2) and third eruption (Obsidian flow) was marked by a phreatomagmatic vent-clearing blast^22^, which would have suddenly increased conduit dimensions, favoring stability conditions and the development of more steady explosive events, in comparison with the two first eruptions. For Inyo Craters, geological evidences strongly support that magma ascent is particularly favored by structural settings^23–25^, thus it seems difficult to use lithic content to estimate conduit dimensions. Still, even in the latter two eruptions, total volumes of lithic fragments (Supplementary Table S4)^22^ are sensibly lower than that observed in sustained rhyolitic eruptions and thus much narrower, unstable conduits are expected. An additional case study of pulsating rhyolitic eruptions is the latest explosive eruption in Mono Craters^23^, characterized by low mass discharge rates and low volumes of lithic fragments, suggesting that they were unable to develop wide, stable feeding conduits.

## Concluding remarks

Modeling of magma ascent through axisymmetric volcanic conduits with fixed and depth-dependent radii suggests that magma composition and rheology play a leading role in defining the conditions of conduit stability, and consequently in controlling conduit dimensions, evolution of mass discharge rate and the amount of lithic fragments found in pyroclastic deposits. In particular:

- Dacitic/rhyolitic magmas need conduits several times wider than phonolitic/trachytic magmas for developing mechanically stable conduits, which could explain the high mass discharge rates commonly observed in sustained dacitic/rhyolitic eruptions, whereas phonolitic/trachytic magmas would be capable of producing sustained explosive eruptions with relatively low mass discharge rates.
- Conduit stability can influence the steadiness of volcanic eruptions, controlling the occurrence of pulsating variations in the eruptive parameters. Narrow, unstable conduits would be commonly related with oscillating volcanic eruptions, whereas wide, stable feeding conduits could produce steady explosive events and homogeneous pyroclastic deposits.
- Conduit wall collapses seem to be particularly dominant during inception/waxing phases of explosive events, determining whether a well-developed steady-state phase is reached and thus controlling the early dynamics of volcanic events and the viability of sustained eruptions. Conduit collapse is also likely to occur during the waning phases of explosive eruptions, because of the expected inlet pressure drop, generating conduit blockages and controlling the eruption shutdown.
- The nature of the erosive process along the conduit during volcanic eruptions is a first order factor for controlling the resulting eruptive dynamics, modifying parameters such as mass discharge rate, exit pressure and fragmentation depth. In particular, the eruptive dynamics would be conditioned by the ability of the conduit to inhibit widening processes above the fragmentation level.

## Methods

In order to study the pressure field along volcanic conduits (P(z)), we employed a 1D multiphase steady-state model which considers the main processes experienced by magmas during their ascent, such as crystallization, rheological changes, magma fragmentation, interaction with conduit walls, outgassing and degassing. We used appropriate sets of constitutive equations for describing the physical parameters of the system (Supplementary Table S5), chosen for studying representative cases of phonolitic, trachytic, dacitic and rhyolitic explosive volcanism. Initially, we considered a 5000-m long axisymmetric conduit with fixed dimensions in depth, and varied three input parameters: inlet pressure, water content and conduit radius (Supplementary Tables S5 and S6).

For evaluating the mechanical stability of the conduit, we calculated the minimum pressure needed to avoid conduit collapse (P_collapse_(z)), as described in Aravena *et al*.^8^. For that, we applied the Mogi-Coulomb criterion presented by Al-Ajmi and Zimmerman^10^ (Supplementary Table S7), by using the mechanical parameters shown in Supplementary Table S8. With the objective of quantifying the instability character of the conduit, we compared P_collapse_(z) and P(z), using the following expression, called instability index^8^:

1 $${\rm{\max }}({{\rm{P}}}_{{\rm{collapse}}}({\rm{z}})-{\rm{P}}({\rm{z}}))$$

Since a monotonic relationship is observed between conduit radius and instability index^8^, for given conditions of water content and inlet pressure, we interpolated the radius at which the instability index is equal to zero (critical radius), using a second order fit. Accordingly, the critical radius represents the minimum radius needed to avoid conduit collapse, for a given set of magma source conditions.

Critical radius calculations were also employed for studying the temporal evolution of volcanic conduits by assuming axisymmetric geometries with fixed radius in depth, whose dimensions are continuously re-equilibrated to the changing stability conditions produced by modifications of inlet pressure and water content, for trachytic and rhyolitic magmas. Inlet pressure was assumed to vary linearly between two fixed limits (135 MPa and 115 MPa), as a function of the mass of erupted magma; considering a total erupted mass of 10^11^ kg and 2·10^12^ kg for trachytic and rhyolitic magmas, respectively. This assumption is supported in the absence of exsolved water in magma reservoir for the solubility models here adopted (conduit length of 5000 m), and thus nearly incompressible melts are expected to fill the magma reservoir. For a given magma composition, two different hypothetical events were modelled, which are characterized by two different prescribed mathematical relations between inlet pressure and water content, and reflect discrepancies in compositional zonations of magma chamber (Fig. 4a and e). Still, initial and final input parameters are coincident for both modelled trends of a given magma composition, while the total eruption time is controlled by the resulting eruption rate and total erupted mass. In order to calculate the expected content of lithic fragments in pyroclastic deposits, we analyzed the modifications of conduit dimensions as critical radius changes.

For testing the effects of different geometries, we compared four cases: (1) axisymmetric conduits with fixed radius in depth (geometry C), (2) axisymmetric fixed-radius conduits with a local enlargement along a vertical distance z_r_ (geometry NC1), (3) conduits with two coaxial cylindrical portions connected by a linearly variable transitional zone of length z_r_ (geometry NC2) and (4) conduits with an axisymmetric lower portion with fixed radius in depth, connected with the surface by a linearly enlarging zone (geometry NC3). In simulations of axisymmetric conduits with depth-dependent radius (i.e. geometries NC1, NC2 and NC3), we set the variable-radius zone (z_c_) around the fragmentation level computed in the fixed-radius equivalent case (i.e. R_fixed_(C) = R_min_(NC), with the same values of inlet pressure and water content); varying R_max_/R_min_ and z_r_ between specific limits (Supplementary Table S6).

On the other hand, in order to compare the typical intensity of explosive volcanic eruptions related to different magma compositions, we used information derived from a global database (LaMEVE)^26^. As an intensity measure, we employed the eruptive column height^11^, splitting data into four subsets: (1) basaltic and andesitic basaltic magmas, (2) andesitic magmas, (3) dacitic and rhyolitic magmas and (4) phonolitic and trachytic magmas. When two or more results are reported for different phases of a unique eruption, we selected the highest value; and we discarded all data with other compositions or information difficult to split (e.g. dacitic andesite, trachyandesite). Descriptive statistics and variance analysis were employed for results interpretation.

## Electronic supplementary material

Supplementary material
