# Supplementary material for: Conduit stability effects on intensity and steadiness of explosive eruptions
Source: Sci Rep. 2018 Mar 7;8:4125. doi: 10.1038/s41598-018-22539-8 (PMC5841421; doi:10.1038/s41598-018-22539-8)
Supplement: Supplementary file 1 — Supplementary material [file 41598_2018_22539_MOESM1_ESM.pdf]

Conduit stability effects on intensity and steadiness of explosive eruptions

Aravena, Álvaro[1*]; Cioni, Raffaello[1]; de'Michieli Vitturi, Mattia[2]; Neri, Augusto[2].

1 Dipartimento di Scienze della Terra, Università di Firenze, Firenze, Italy.

2 Istituto Nazionale di Geofisica e Vulcanologia, Sezione di Pisa, Pisa, Italy.

Correspondence to: alvaro.aravenaponce@unifi.it

Supplementary material

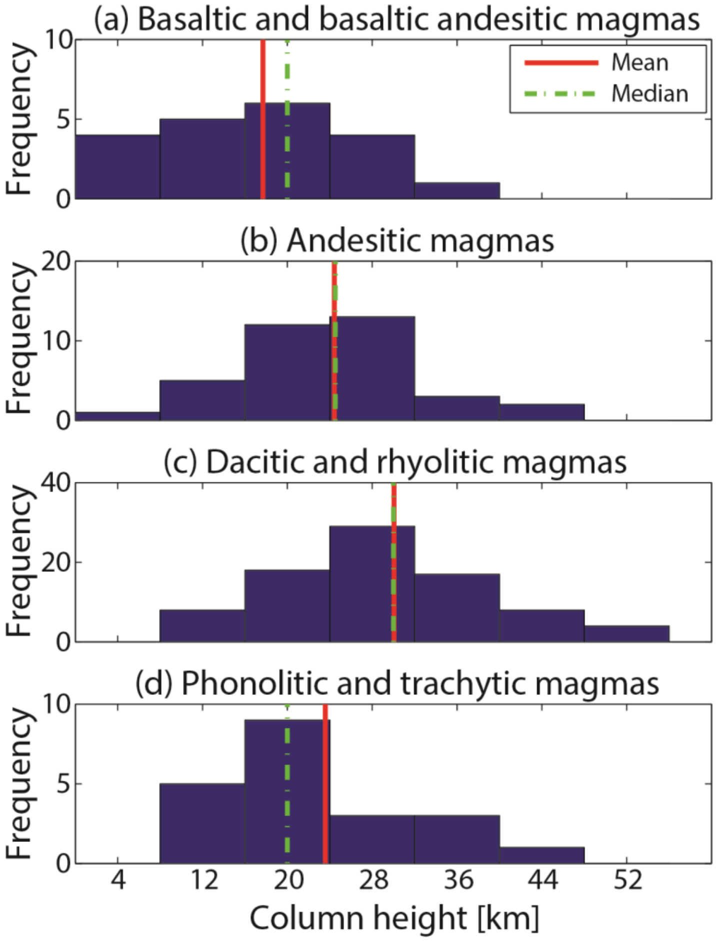

Figure S1. Histograms of eruptive column height of a set of documented explosive volcanic eruptions, for different magma compositions. Data have been extracted from the database LaMEVE[1]. (a) Basaltic and basaltic andesitic magmas (N=20). (b) Andesitic magmas (N=36). (c) Dacitic and rhyolitic magmas (N=84). (d) Phonolitic and trachytic magmas (N=21). Statistical parameters are shown in supplementary Table S1.

Table S1. Statistical parameters of the eruptive column height distributions reported in supplementary Figure S1, using a set of documented explosive volcanic eruptions, for different magma compositions. Data have been extracted from the database LaMEVE[1]. As a referential information, the corresponding values of MDR are also reported, considering the average values of column height, absence of wind and the results presented in de'Michieli Vitturi et al.[2].

**Statistical parameters of column heights**	**Basaltic and basaltic andesitic magmas**	**Andesitic magmas**	**Rhyolitic and dacitic magmas**	**Phonolitic and trachytic magmas**
Mean (km)	17.7	24.4	30.1	23.6
Median (km)	20.0	24.5	30.0	20.0
Mode (km)	20.0	28.0	30.0	20.0
Skewness	-0.11	0.13	0.50	0.68
Kurtosis	1.99	3.07	3.02	2.36
Correspondent mean MDR (kg/s)	$3.7 \cdot 10^{7}$	$1.7 \cdot 10^{8}$	$4.7 \cdot 10^{8}$	$1.5 \cdot 10^{8}$

Table S2. Summary of results derived from a variance analysis of the eruptive column height, considering the interrelations between four different groups (basaltic/basaltic andesitic magmas; andesitic magmas; dacitic/rhyolitic magmas; and phonolitic/trachytic magmas). Our results indicate that dacitic/rhyolitic magmas present average values of eruptive column height significantly different from phonolitic/trachytic magmas and all the other compositional groups, using a confidence interval of 95% (i.e. p-values lower than 0.05). Data have been extracted from the database LaMEVE[1].

**Group 1**	**Group 2**	**Lower bound of their difference**	**Estimate of their difference**	**Upper bound of their difference**	**p-value**
Basaltic/Basaltic andesitic	Andesitic	-13.9	-6.7	0.4	0.0117
Basaltic/Basaltic andesitic	Dacitic/Rhyolitic	-18.8	-12.4	-6.0	$\mathbf{1.3 \cdot 10^{-6}}$
Basaltic/Basaltic andesitic	Phonolitic/Trachytic	-13.9	-5.9	2.2	0.0637
Andesitic	Dacitic/Rhyolitic	-10.7	-5.6	-0.5	**0.0038**
Andesitic	Phonolitic/Trachytic	-6.2	0.9	7.9	0.7454
Dacitic/Rhyolitic	Phonolitic/Trachytic	0.2	6.4	12.8	**0.0080**

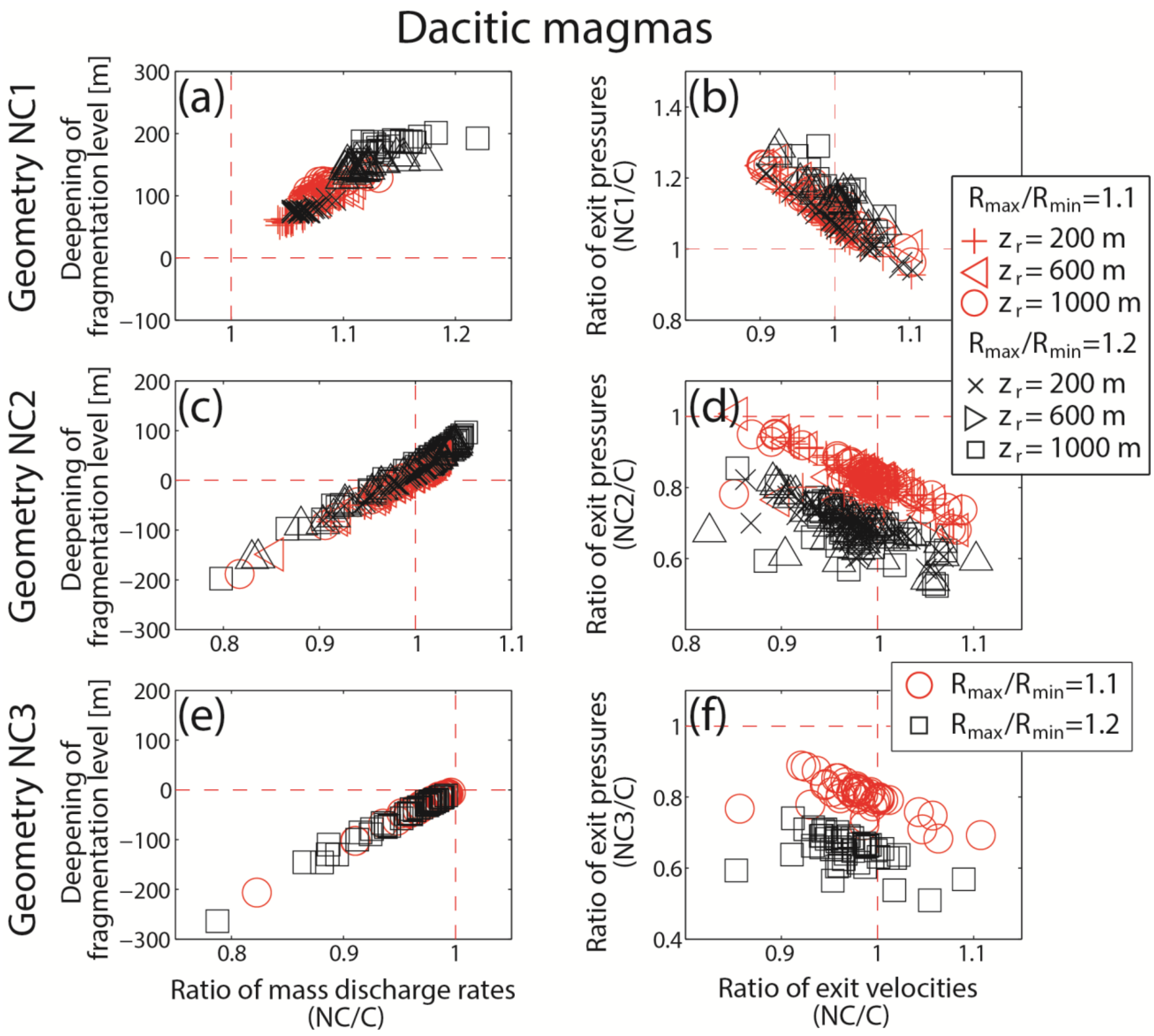

Figure S2. (a) Ratio of mass discharge rates versus deepening of fragmentation level (NC1 versus fixed-radius equivalent case). (b) Ratio of exit velocities versus ratio of exit pressures (NC1 versus fixed-radius equivalent case). (c) Ratio of mass discharge rates versus deepening of fragmentation level (NC2 versus fixed-radius equivalent case). (d) Ratio of exit velocities versus ratio of exit pressures (NC2 versus fixed-radius equivalent case). (e) Ratio of mass discharge rates versus deepening of fragmentation level (NC3 versus the fixed-radius equivalent case). (f) Ratio of exit velocities versus ratio of exit pressures (NC3 versus fixed-radius equivalent case). Here we present results related to dacitic magmas.

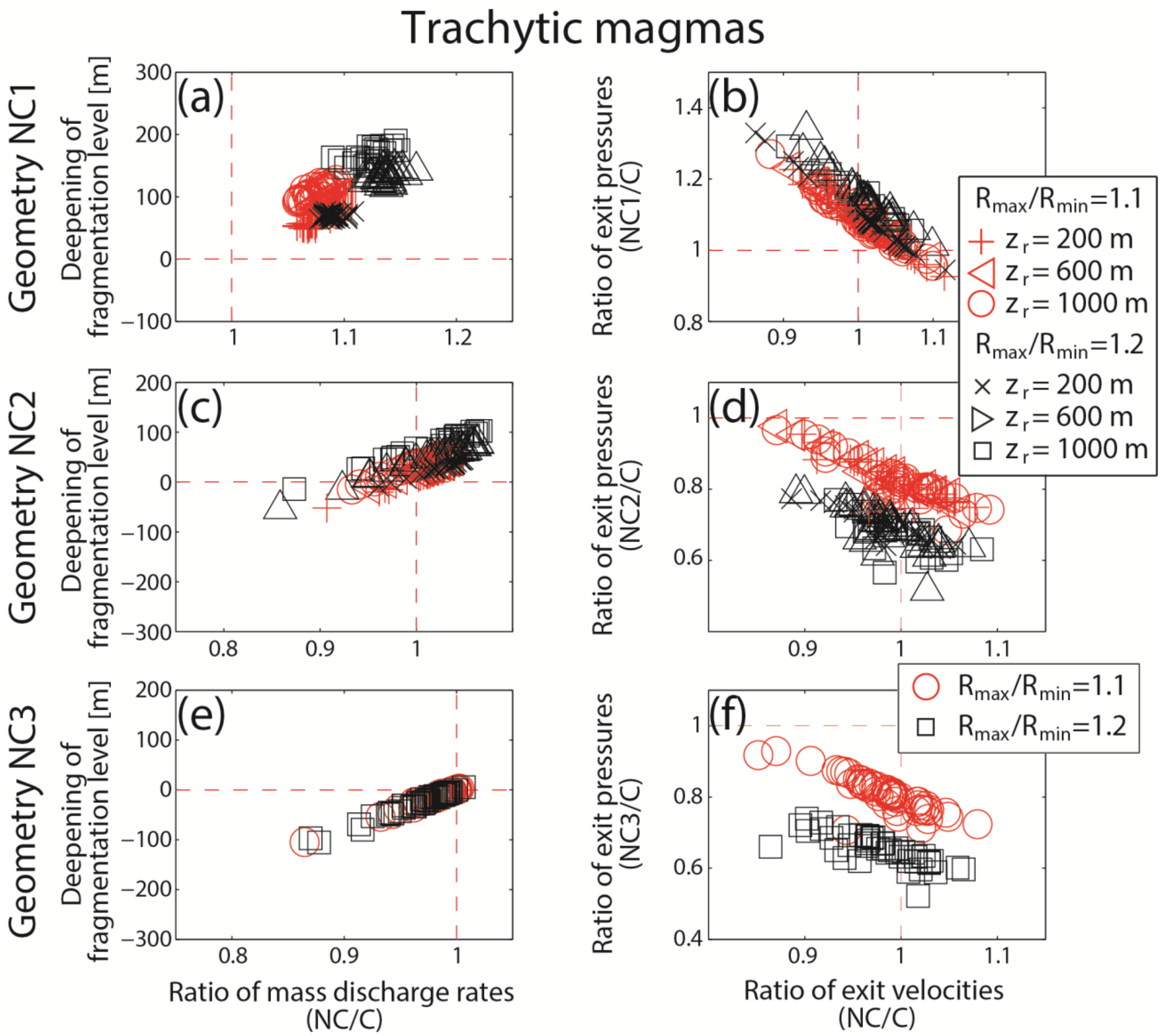

Figure S3. (a) Ratio of mass discharge rates versus deepening of fragmentation level (NC1 versus fixed-radius equivalent case). (b) Ratio of exit velocities versus ratio of exit pressures (NC1 versus fixed-radius equivalent case). (c) Ratio of mass discharge rates versus deepening of fragmentation level (NC2 versus fixed-radius equivalent case). (d) Ratio of exit velocities versus ratio of exit pressures (NC2 versus fixed-radius equivalent case). (e) Ratio of mass discharge rates versus deepening of fragmentation level (NC3 versus fixed-radius equivalent case). (f) Ratio of exit velocities versus ratio of exit pressures (NC3 versus fixed-radius equivalent case). Here we present results related to trachytic magmas.

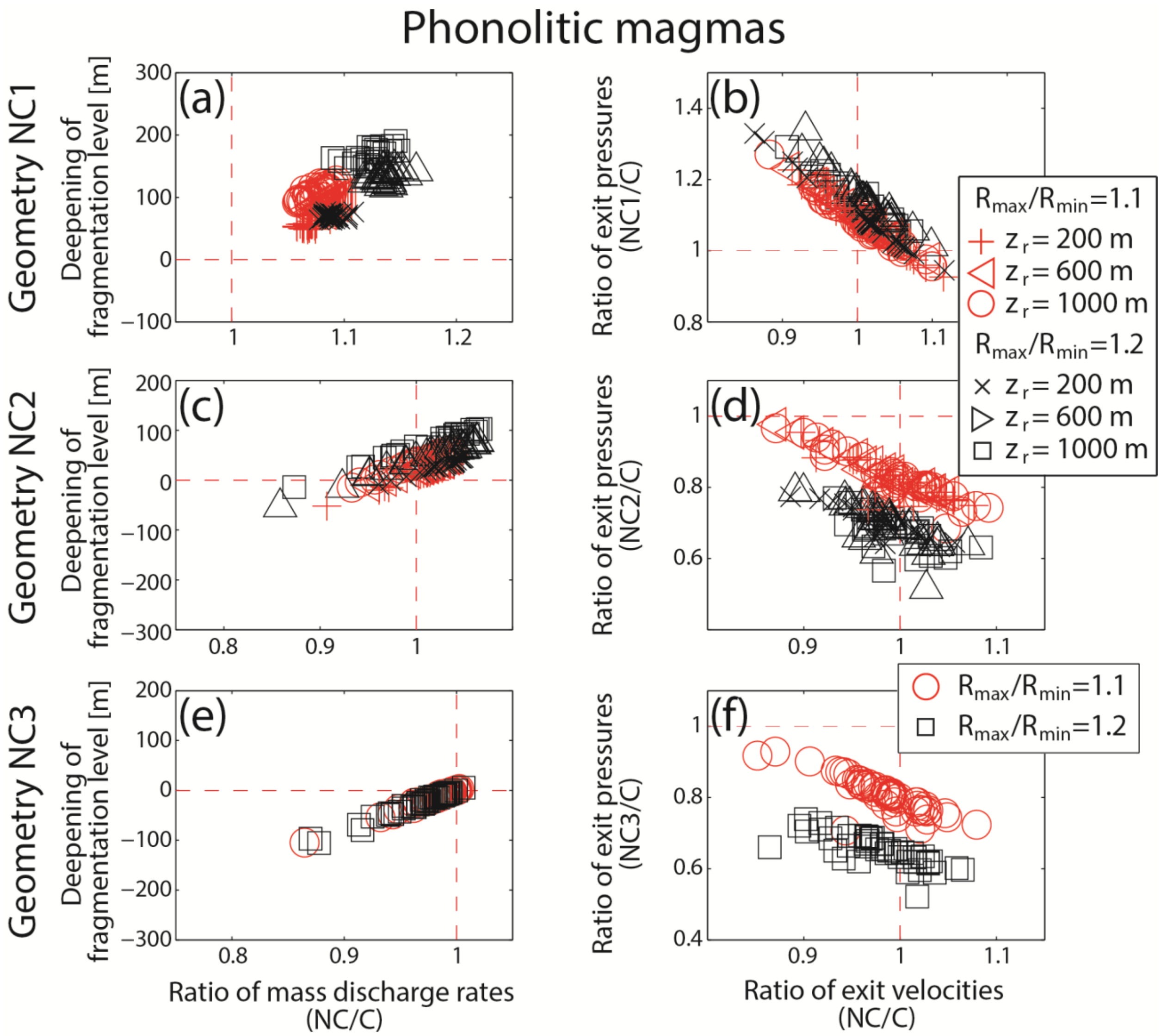

Figure S4. (a) Ratio of mass discharge rates versus deepening of fragmentation level (NC1 versus fixed-radius equivalent case). (b) Ratio of exit velocities versus ratio of exit pressures (NC1 versus fixed-radius equivalent case). (c) Ratio of mass discharge rates versus deepening of fragmentation level (NC2 versus fixed-radius equivalent case). (d) Ratio of exit velocities versus ratio of exit pressures (NC2 versus fixed-radius equivalent case). (e) Ratio of mass discharge rates versus deepening of fragmentation level (NC3 versus fixed-radius equivalent case). (f) Ratio of exit velocities versus ratio of exit pressures (NC3 versus fixed-radius equivalent case). Here we present results related to phonolitic magmas.

Table S3. Some eruption parameters for units identified in the Mangaone Subgroup[3].

Unit	Total mass (kg)	Peak mass discharge rate (kg/s)
A	$3.0 \cdot 10^{11}$	-
B	$2.8 \cdot 10^{12}$	$1.9 \cdot 10^{8}$
C	$4.3 \cdot 10^{11}$	$8.6 \cdot 10^{7}$
D	$6.5 \cdot 10^{12}$	$2.7 \cdot 10^{8}$
E	$8.0 \cdot 10^{11}$	$1.2 \cdot 10^{8}$
F	$9.3 \cdot 10^{12}$	$3.3 \cdot 10^{8}$
G	$1.5 \cdot 10^{12}$	$1.4 \cdot 10^{8}$
H	$2.5 \cdot 10^{11}$	-
I	$1.5 \cdot 10^{13}$	$3.0 \cdot 10^{8}$
J	$5.5 \cdot 10^{11}$	$6.2 \cdot 10^{7}$
K	$9.8 \cdot 10^{12}$	$3.9 \cdot 10^{8}$
L	$3.2 \cdot 10^{12}$	$2.0 \cdot 10^{8}$

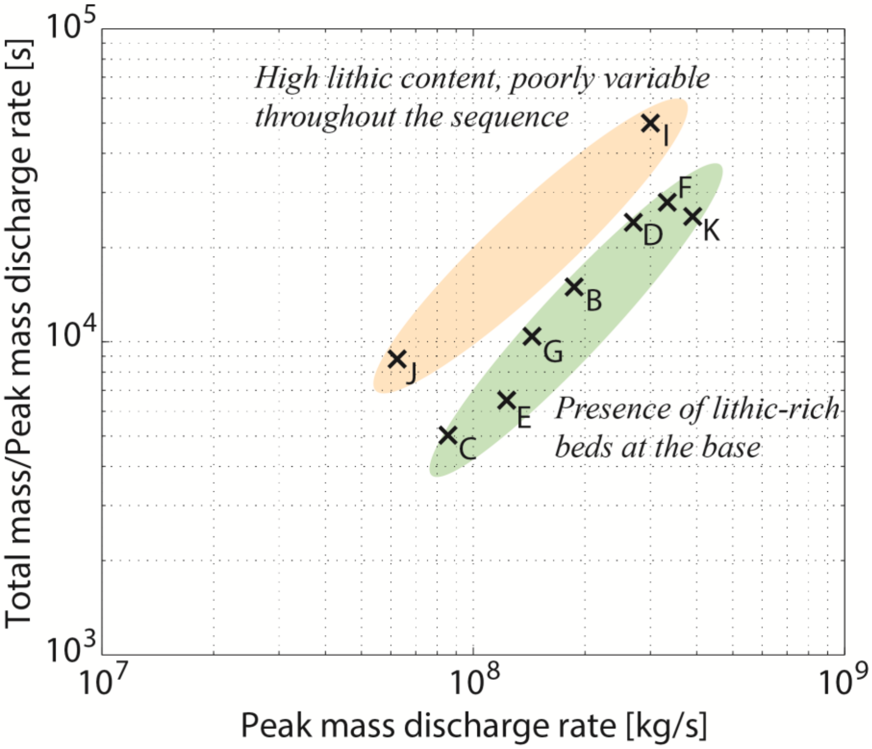

Figure S5. Ratio between total erupted mass and peak mass discharge rate versus peak mass discharge rate, for units identified in the Mangaone Subgroup[3]. For clarity, unit L was not included in this figure because it is characterized by the presence of deposits of pyroclastic flows at the base, and thus it is difficult to evaluate the variation trends of lithic fragments in the eruptive sequence (indeed, this is the only case where more than 35% of the erupted mass corresponds to deposits of PDCs). Still, in this figure, unit L would be located near the limit between both fields, and their fallout deposits are characterized by high lithic contents and non-monotonic variation trends.

Table S4. Some parameters of the eruption identified in Inyo Craters[4,5]. ([a]) Based on lithic fragments-derived estimations. ([b]) We assume a lithic fragments density of 2600 kg/m^3 and a pumice density of 600 kg/m^3. ([c]) Including obsidian fragments. ([d]) Lower Obsidian Flow. ([e]) Upper Obsidian Flow.

Eruption	Total volume (km^3)	Mass discharge rate (kg/s) ([a])	Mass percentage of lithics	Total volume of lithic fragments (m^3) ([b])	Steadiness
South Deadman 1	0.01	$1 \cdot 10^6$ - $4 \cdot 10^6$	10% ([c])	$3.2 \cdot 10^5$	Highly pulsating sub-Plinian column
South Deadman 2	0.04	$2 \cdot 10^6$ - $1 \cdot 10^7$	15-22% ([c])	$2.1 \cdot 10^6$ - $3.6 \cdot 10^6$	Highly pulsating sub-Plinian column
Obsidian Flow	<0.01 (OFL([d]))<br>0.01-0.02 (OFU([e]))	$3 \cdot 10^7$	60% (OFL)<br>25% (OFU)	$4.2 \cdot 10^6$ (OFL)<br>$1.1 \cdot 10^6$ - $2.2 \cdot 10^6$ (OFU)	Quasi-sustained sub-Plinian column
Glass Creek	0.10	$5 \cdot 10^6$ - $4 \cdot 10^7$	10%	$3.2 \cdot 10^6$	Quasi-sustained sub-Plinian column

Table S5. Magma parameters and models used in numerical simulations. ([a]) Based on Carroll and Blank[6] (T2-182). ([b]) Based on Di Matteo et al.[7] (SPAT). ([c]) Based on Carroll and Blank[6]. ([d]) Based on Di Matteo et al. (ZAC)[7]. ([e]) Based on Moore[8]. ([f]) Based on Zhang[9].

Property	Phonolitic magma	Trachytic magma	Dacitic magma	Rhyolitic magma
Temperature	900ºC	900ºC	850ºC	850ºC
Viscosity model	Giordano et al.[10] ([a])	Giordano et al.[10] ([b])	Whittington et al.[11]	Hess and Dingwell[12]
Influence of crystals on viscosity	Costa[13]	Costa[13]	Costa[13]	Costa[13]
Influence of bubbles on viscosity	Costa et al.[14]	Costa et al.[14]	Costa et al.[14]	Costa et al.[14]
Exsolved gas model	Ideal gas	Ideal gas	Ideal gas	Ideal gas
Crystallinity model	de'Michieli Vitturi et al.[15]	de'Michieli Vitturi et al.[15]	de'Michieli Vitturi et al.[15]	de'Michieli Vitturi et al.[15]
Solubility model	Polynomial fit ([c])	Polynomial fit ([d])	Henri's law ([e])	Henri's law ([f])
Inlet pressure	115 – 135 MPa	115 – 135 MPa	115 – 135 MPa	115 – 135 MPa
Water content	4.0 – 6.0 wt.%	4.0 – 6.0 wt.%	4.5 – 6.5 wt.%	4.5 – 6.5 wt.%

Table S6. Geometric parameters used in numerical simulations. ([a]) Some additional simulations have been performed in order to improve the quality of the interpolations of critical radius.

Geometric parameter	Geometry C	Geometries NC1/NC2/NC3
Fixed radius ([a])	5 – 50 m (phonolitic magma)<br>5 – 50 m (trachytic magma)<br>7.5 - 60 m (dacitic magma)<br>30 - 110 m (rhyolitic magma)	-
R_{min}	-	10 – 50 m (phonolitic magma)<br>10 – 50 m (trachytic magma)<br>10 – 50 m (dacitic magma)<br>40 – 90 m (rhyolitic magma)
R_{max}/R_{min}	-	1.1 – 1.2
z_c	-	Fragmentation level of the fixed-radius equivalent case ($R = R_{min}$)
z_r	-	200 m – 1000 m

Table S7. Mogi-Coulomb criterion for collapse pressure[16].
$P_{collapse}$: Minimum pressure for inhibiting conduit collapse.
σ_z: Vertical stress. σ_r: Radial stress. σ_θ: Tangential stress.
$a' = 2c \cdot \cos(\phi)$. $b' = \sin(\phi)$. c: Cohesion. ϕ: Angle of internal friction.
$A = 3\sigma_H - \sigma_h$. σ_H: Maximum horizontal stress. σ_h: Minimum horizontal stress.
$B = \sigma_v + 2\upsilon(\sigma_H - \sigma_h)$. σ_v: Vertical stress. υ: Poisson ratio.
$G = K + b'A$. $H = A^2(4b'^2 - 3) + (B^2 - AB)(4b'^2 - 12)$. $K = a' + b'(B - 2P_0)$. P_0: Pore pressure.

Case	$\sigma_1 \geq \sigma_2 \geq \sigma_3$	Collapse occurs if $P \geq P_{collapse}$
1	$\sigma_z \geq \sigma_\theta \geq \sigma_r$	$P_{collapse} = \frac{1}{6-2b'^2} \cdot \left[(3A + 2b'K) - \sqrt{H + 12(K^2 + b'AK)}\right]$
2	$\sigma_\theta \geq \sigma_z \geq \sigma_r$	$P_{collapse} = \frac{A}{2} - \frac{1}{6}\sqrt{12[a' + b'(A - 2P_0)]^2 - 3(A - 2B)^2}$
3	$\sigma_\theta \geq \sigma_r \geq \sigma_z$	$P_{collapse} = \frac{1}{6-2b'^2} \cdot \left[(3A - 2b'G) - \sqrt{H + 12(G^2 + b'AG)}\right]$

Table S8. Parameters related to stability criteria, representative of typical conditions[17].

Parameter	Value
Rock cohesion	5 MPa
Angle of friction	38°
Vertical stress gradient	26 kPa/m
Both horizontal stress gradients	18 kPa/m

Supplementary material references:

1. Crosweller, H. S. *et al.* Global database on large magnitude explosive volcanic eruptions (LaMEVE). *Journal of Applied Volcanology* **1**, 1-13 (2012).
2. de'Michieli Vitturi, M., Engwell, S., Neri, A. & Barsotti, S. Uncertainty quantification and sensitivity analysis of volcanic columns models: Results from the integral model PLUME-MoM. *Journal of Volcanology and Geothermal Research* **326**, 77-91 (2016).
3. Jurado-Chichay, Z. & Walker, G. The intensity and magnitude of the Mangaone subgroup plinian eruptions from Okataina Volcanic Centre, New Zealand. *Journal of volcanology and geothermal research* **111**, 219-237 (2001).
4. Nawotniak, S. K. & Bursik, M. Subplinian fall deposits of Inyo Craters, CA. *Journal of Volcanology and Geothermal Research* **198**, 433-446 (2010).
5. Miller, C. D. Holocene eruptions at the Inyo volcanic chain, California: Implications for possible eruptions in Long Valley caldera. *Geology* **13**, 14-17 (1985).
6. Carroll, M. R. & Blank, J. G. The solubility of H2O in phonolitic melts. *American Mineralogist* **82**, 549-556 (1997).
7. Di Matteo, V., Carroll, M., Behrens, H., Vetere, F. & Brooker, R. Water solubility in trachytic melts. *Chemical Geology* **213**, 187-196 (2004).
8. Moore, G., Vennemann, T. & Carmichael, I. An empirical model for the solubility of H2O in magmas to 3 kilobars. *american Mineralogist* **83**, 36-42 (1998).
9. Zhang, Y. H2O in rhyolitic glasses and melts: measurement, speciation, solubility, and diffusion. *Reviews of Geophysics* **37**, 493-516 (1999).
10. Giordano, D., Russell, J. K. & Dingwell, D. B. Viscosity of magmatic liquids: a model. *Earth and Planetary Science Letters* **271**, 123-134 (2008).
11. Whittington, A. G. *et al.* The viscosity of hydrous dacitic liquids: implications for the rheology of evolving silicic magmas. *Bulletin of Volcanology* **71**, 185-199 (2009).
12. Hess, K. & Dingwell, D. Viscosities of hydrous leucogranitic melts: A non-Arrhenian model. *American Mineralogist* **81**, 1297-1300 (1996).
13. Costa, A. Viscosity of high crystal content melts: dependence on solid fraction. *Geophysical Research Letters* **32** (2005).
14. Costa, A., Melnik, O., Sparks, R. & Voight, B. Control of magma flow in dykes on cyclic lava dome extrusion. *Geophysical Research Letters* **34** (2007).
15. de'Michieli Vitturi, M., Clarke, A., Neri, A. & Voight, B. Transient effects of magma ascent dynamics along a geometrically variable dome-feeding conduit. *Earth and Planetary Science Letters* **295**, 541-553 (2010).
16. Al-Ajmi, A. M. & Zimmerman, R. W. Stability analysis of vertical boreholes using the Mogi–Coulomb failure criterion. *International Journal of Rock Mechanics and Mining Sciences* **43**, 1200-1211 (2006).
17. Hoek, E. & Brown, E. Practical estimates of rock mass strength. *International Journal of Rock Mechanics and Mining Sciences* **34**, 1165-1186 (1997).
